# Bis{2-formyl-4-methyl-6-[(tri-2-pyridyl­meth­yl)imino­meth­yl]phenolato}nickel(II)

**DOI:** 10.1107/S160053680900244X

**Published:** 2009-01-28

**Authors:** Yan-Qiu Dang, Hong-Jun Yang, Lai-Jin Tian

**Affiliations:** a Department of Chemistry and Chemical Engineering, Binzhou University, Binzhou 256600, People’s Republic of China; b Research Center for Eco-Environmental Sciences of the Yellow River Delta, Binzhou University, Binzhou 256600, People’s Republic of China; c Department of Chemistry, Qufu Normal University, Qufu 273165, People’s Republic of China

## Abstract

The title compound, [Ni(C_25_H_19_N_4_O_2_)_2_], which was obtained by the reaction of nickel(II) perchlorate with 2,6-diformyl-4-methyl­phenol and (tri-2-pyridylmeth­yl)amine in methanol solution, is a discrete monometallic complex. The Ni^II^ atom is six-coordinated by the phenolate O, imine N and pyridine N atoms from two tridentate Schiff base ligands in a distorted NiN_4_O_2_ octa­hedral geometry. The dihedral angles between the noncoordinated pyridyl rings of each ligand are 72.95 (8) and 69.59 (7)°.

## Related literature

For related structures, see: Arnold *et al.* (2003 ▶); Cumming *et al.* (1977 ▶); Manonmani *et al.* (2001 ▶); Parker *et al.* (2007 ▶); Li & Gao (2007 ▶); Tian *et al.* (2007 ▶). For background, see: Borisova *et al.* (2007 ▶); Bruckner *et al.* (2000 ▶).
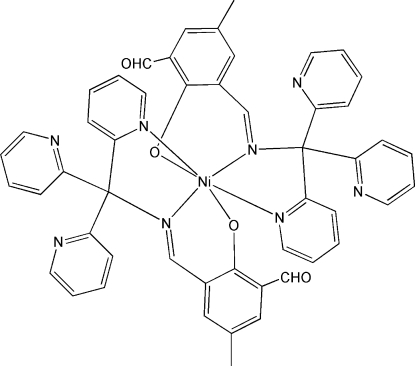


## Experimental

### 

#### Crystal data

[Ni(C_25_H_19_N_4_O_2_)_2_]
*M* *_r_* = 873.59
Monoclinic, 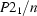

*a* = 11.9592 (2) Å
*b* = 17.6301 (2) Å
*c* = 19.6633 (3) Å
β = 98.202 (1)°
*V* = 4103.44 (10) Å^3^
*Z* = 4
Mo *K*α radiation
μ = 0.53 mm^−1^
*T* = 295 (2) K
0.22 × 0.16 × 0.04 mm

#### Data collection

Bruker APEX CCD diffractometer
Absorption correction: multi-scan (*SADABS*; Bruker, 2002 ▶) *T*_min_ = 0.892, *T*_max_ = 0.979
45290 measured reflections
8063 independent reflections
6167 reflections with *I* > 2σ(*I*)
*R*_int_ = 0.041

#### Refinement

*R*[*F* ^2^ > 2σ(*F* ^2^)] = 0.036
*wR*(*F* ^2^) = 0.095
*S* = 1.02
8063 reflections
570 parameters
H-atom parameters constrained
Δρ_max_ = 0.29 e Å^−3^
Δρ_min_ = −0.29 e Å^−3^

### 

Data collection: *SMART* (Bruker, 2002 ▶); cell refinement: *SAINT* (Bruker, 2002 ▶); data reduction: *SAINT*; program(s) used to solve structure: *SHELXS97* (Sheldrick, 2008 ▶); program(s) used to refine structure: *SHELXL97* (Sheldrick, 2008 ▶); molecular graphics: *ORTEP-3 for Windows* (Farrugia, 1997 ▶); software used to prepare material for publication: *SHELXL97*.

## Supplementary Material

Crystal structure: contains datablocks global, I. DOI: 10.1107/S160053680900244X/hb2899sup1.cif

Structure factors: contains datablocks I. DOI: 10.1107/S160053680900244X/hb2899Isup2.hkl

Additional supplementary materials:  crystallographic information; 3D view; checkCIF report

## Figures and Tables

**Table 1:** Selected bond lengths (Å)

Ni1—N3	2.0210 (17)
Ni1—O2	2.0283 (13)
Ni1—N1	2.0318 (17)
Ni1—O1	2.0322 (13)
Ni1—N2	2.1084 (16)
Ni1—N4	2.1308 (16)

**Fig. 1.**
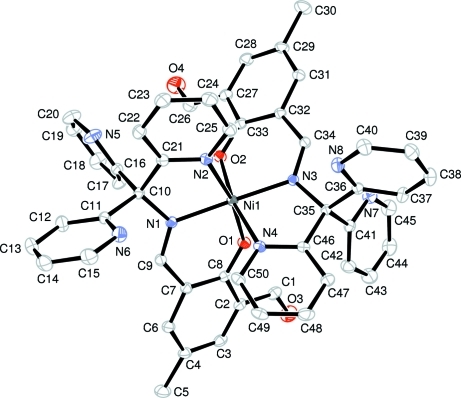
The molecular structure of (I) with displacement ellipsoids drawn at the 30% probability level. H atoms have been omitted for clarity.

## supplementary crystallographic information

### Comment

Nickel complexes with Schiff bases have received much attention in recent years due to their pharmacological and catalytic properties (Borisova *et al.*, 2007; Bruckner *et al.*, 2000). 2-(Tri-2-pyridylmethyliminomethyl)phenol is a potential N_4_O pentadentate Schiff base ligand and its complexes with copper(II) and nickel(II) have been reported (Arnold *et al.*, 2003; Li & Gao, 2007; Tian *et al.*, 2007). As a continuation of the work, the synthesis and structure of the title compound, (I), now are discussed.

In this mononuclear nickel complex, the environment around the central nickel atom is a distorted octahedron (Table 1) with the three axial bond angles being 173.34 (6), 168.45 (6) and 168.03 (6)°. The two Schiff base ligands are both tridentate and coordinated in a meridional fashion. Each ligand coordinates to the metal by an NN'O donor set *via* an imine N, one pyridine N and a phenolate O. The other two N atoms of the pyridine rings in each Schiff base ligand are distant from the metal. The meridional coordination of each ligand leads to *cis* orientations of the two coordinated pyridine rings and two phenolate rings, and the *trans* orientation of two imine N atoms. The Ni1—N2 and Ni1—N4 (pyridine) distances are appreciably longer than those for Ni1—N1 and Ni1—N3 (imine), which is agreement with those of azido{2-[(tri-2-pyridylmethylimino)methyl]phenolato}nickel(II) (Tian *et al.*, 2007). The Ni1—O1 and Ni1—O2 distances are almost the same as those found in the six-coordinated NiN_4_O_2_ complexes such as [*N*,*N*'-bis(2-Salicylideneaminoethyl)ethylenediamine]nickel(II) (Cumming *et al.*, 1977), {*N*,*N*'-bis[2-Hydroxy-3-(1-morpholinioylmethyl)-5-\ methylbenzylidene] triethylenetetraamine}nickel(II) (Manonmani *et al.*, 2001), and bis{2,4-dibutyl-6-[2-(dimethylamino)ethyliminomethyl]phenolato}nickel(II) (Parker *et al.*, 2007).

### Experimental

2,6-Diformyl-4-methylphenol (0.164 g, 1 mmol), tri-2-pyridylmethylamine (0.262 g, 1 mmol) and Ni(ClO_4_)_2_.6H_2_O (0.183 g, 0.5 mmol) were stirred in methanol (20 ml) for 20 min at room temperature, and then filtered. After keeping the filtrate in air for 3 d, green plates of (I) (yield 36%) were formed.

### Refinement

The H atoms were placed at calculated positions and refined in the riding-model approximation, with C—H = 0.93 Å and *U*_iso_(H) = 1.2Ueq(C) for aromatic and formyl H atoms, and C—H = 0.96 Å and *U*_iso_(H) = 1.5Ueq(C) for methyl H atoms.

### Crystal data

**Table:**

[Ni(C_25_H_19_N_4_O_2_)_2_]	*F*(000) = 1816
*M**_r_* = 873.59	*D*_x_ = 1.414 Mg m^−^^3^
Monoclinic, *P*2_1_/*n*	Mo *K*α radiation, λ = 0.71073 Å
Hall symbol: -P 2yn	Cell parameters from 5403 reflections
*a* = 11.9592 (2) Å	θ = 2.4–24.2°
*b* = 17.6301 (2) Å	µ = 0.53 mm^−^^1^
*c* = 19.6633 (3) Å	*T* = 295 K
β = 98.202 (1)°	Plate, green
*V* = 4103.44 (10) Å^3^	0.22 × 0.16 × 0.04 mm
*Z* = 4	

### Data collection

**Table:**

Bruker APEX CCD diffractometer	8063 independent reflections
Radiation source: fine-focus sealed tube	6167 reflections with *I* > 2σ(*I*)
graphite	*R*_int_ = 0.041
φ and ω scans	θ_max_ = 26.0°, θ_min_ = 1.6°
Absorption correction: multi-scan (*SADABS*; Bruker, 2002)	*h* = −13→14
*T*_min_ = 0.892, *T*_max_ = 0.979	*k* = −21→21
45290 measured reflections	*l* = −24→24

### Refinement

**Table:**

Refinement on *F*^2^	Primary atom site location: structure-invariant direct methods
Least-squares matrix: full	Secondary atom site location: difference Fourier map
*R*[*F*^2^ > 2σ(*F*^2^)] = 0.036	Hydrogen site location: inferred from neighbouring sites
*wR*(*F*^2^) = 0.095	H-atom parameters constrained
*S* = 1.02	*w* = 1/[σ^2^(*F*_o_^2^) + (0.0424*P*)^2^ + 1.7711*P*] where *P* = (*F*_o_^2^ + 2*F*_c_^2^)/3
8063 reflections	(Δ/σ)_max_ = 0.001
570 parameters	Δρ_max_ = 0.29 e Å^−^^3^
0 restraints	Δρ_min_ = −0.29 e Å^−^^3^

### Special details

**Table:**

Geometry. All e.s.d.'s (except the e.s.d. in the dihedral angle between two l.s. planes) are estimated using the full covariance matrix. The cell e.s.d.'s are taken into account individually in the estimation of e.s.d.'s in distances, angles and torsion angles; correlations between e.s.d.'s in cell parameters are only used when they are defined by crystal symmetry. An approximate (isotropic) treatment of cell e.s.d.'s is used for estimating e.s.d.'s involving l.s. planes.
Refinement. Refinement of *F*^2^ against ALL reflections. The weighted *R*-factor *wR* and goodness of fit *S* are based on *F*^2^, conventional *R*-factors *R* are based on *F*, with *F* set to zero for negative *F*^2^. The threshold expression of *F*^2^ > σ(*F*^2^) is used only for calculating *R*-factors(gt) *etc*. and is not relevant to the choice of reflections for refinement. *R*-factors based on *F*^2^ are statistically about twice as large as those based on *F*, and *R*- factors based on ALL data will be even larger.

### Fractional atomic coordinates and isotropic or equivalent isotropic displacement parameters (Å^2^)

**Table:**

	*x*	*y*	*z*	*U*_iso_*/*U*_eq_	
Ni1	0.84948 (2)	0.210253 (13)	0.347678 (13)	0.02795 (8)	
N1	0.75587 (14)	0.17976 (9)	0.25737 (8)	0.0301 (4)	
N2	0.73935 (15)	0.12926 (9)	0.38088 (9)	0.0329 (4)	
N3	0.94630 (14)	0.22760 (9)	0.43930 (8)	0.0282 (4)	
N4	0.98414 (15)	0.13255 (9)	0.34153 (8)	0.0308 (4)	
N5	0.45175 (18)	0.17822 (13)	0.26647 (13)	0.0597 (6)	
N6	0.68774 (17)	0.01602 (10)	0.22315 (10)	0.0468 (5)	
N7	1.13568 (17)	0.31964 (10)	0.49582 (10)	0.0468 (5)	
N8	1.02199 (16)	0.10063 (10)	0.52511 (9)	0.0416 (5)	
O1	0.93693 (12)	0.28502 (8)	0.29681 (7)	0.0368 (3)	
O2	0.74379 (12)	0.29381 (8)	0.36981 (7)	0.0348 (3)	
O3	1.15347 (16)	0.43375 (10)	0.23232 (11)	0.0689 (6)	
O4	0.50123 (18)	0.44825 (13)	0.37676 (11)	0.0829 (7)	
C1	1.0856 (2)	0.38899 (13)	0.24974 (14)	0.0468 (6)	
H1	1.0696	0.3923	0.2946	0.056*	
C2	1.02772 (18)	0.33079 (12)	0.20596 (12)	0.0378 (5)	
C3	1.0438 (2)	0.32684 (13)	0.13762 (12)	0.0444 (6)	
H3	1.0953	0.3599	0.1221	0.053*	
C4	0.9870 (2)	0.27619 (14)	0.09137 (12)	0.0441 (6)	
C5	1.0004 (2)	0.27696 (17)	0.01588 (13)	0.0613 (7)	
H5A	0.9638	0.3211	−0.0056	0.092*	
H5B	0.9666	0.2322	−0.0059	0.092*	
H5C	1.0792	0.2781	0.0114	0.092*	
C6	0.91057 (19)	0.22805 (13)	0.11659 (11)	0.0411 (5)	
H6	0.8727	0.1925	0.0868	0.049*	
C7	0.88725 (18)	0.23003 (12)	0.18463 (11)	0.0345 (5)	
C8	0.94957 (17)	0.28117 (11)	0.23330 (11)	0.0326 (5)	
C9	0.79107 (18)	0.18629 (12)	0.19856 (11)	0.0346 (5)	
H9	0.7507	0.1604	0.1618	0.042*	
C10	0.64618 (17)	0.14184 (11)	0.26192 (10)	0.0320 (5)	
C11	0.62185 (18)	0.07697 (11)	0.20947 (11)	0.0340 (5)	
C12	0.5402 (2)	0.08074 (14)	0.15287 (13)	0.0496 (6)	
H12	0.4971	0.1244	0.1438	0.059*	
C13	0.5230 (2)	0.01863 (17)	0.10944 (14)	0.0615 (7)	
H13	0.4682	0.0201	0.0709	0.074*	
C14	0.5873 (2)	−0.04440 (14)	0.12396 (15)	0.0580 (7)	
H14	0.5765	−0.0871	0.0960	0.070*	
C15	0.6681 (2)	−0.04371 (14)	0.18040 (15)	0.0560 (7)	
H15	0.7121	−0.0869	0.1899	0.067*	
C16	0.55105 (19)	0.20156 (12)	0.25089 (11)	0.0359 (5)	
C17	0.5635 (2)	0.27178 (14)	0.22305 (14)	0.0537 (7)	
H17	0.6335	0.2875	0.2128	0.064*	
C18	0.4704 (3)	0.31892 (16)	0.21042 (15)	0.0654 (8)	
H18	0.4774	0.3665	0.1911	0.079*	
C19	0.3696 (2)	0.29620 (17)	0.22595 (15)	0.0641 (8)	
H19	0.3064	0.3274	0.2183	0.077*	
C20	0.3639 (2)	0.22536 (19)	0.25341 (17)	0.0712 (9)	
H20	0.2943	0.2089	0.2637	0.085*	
C21	0.65235 (18)	0.10801 (11)	0.33457 (11)	0.0343 (5)	
C22	0.5709 (2)	0.05736 (13)	0.35058 (13)	0.0469 (6)	
H22	0.5117	0.0425	0.3173	0.056*	
C23	0.5793 (2)	0.02946 (14)	0.41659 (13)	0.0528 (7)	
H23	0.5252	−0.0039	0.4286	0.063*	
C24	0.6686 (2)	0.05143 (14)	0.46456 (13)	0.0498 (6)	
H24	0.6755	0.0333	0.5094	0.060*	
C25	0.7471 (2)	0.10056 (12)	0.44519 (12)	0.0406 (5)	
H25	0.8080	0.1147	0.4775	0.049*	
C26	0.5755 (2)	0.40153 (15)	0.37765 (14)	0.0555 (7)	
H26	0.5880	0.3818	0.3355	0.067*	
C27	0.64642 (19)	0.37361 (12)	0.43790 (12)	0.0397 (5)	
C28	0.6310 (2)	0.39914 (13)	0.50292 (13)	0.0473 (6)	
H28	0.5741	0.4343	0.5063	0.057*	
C29	0.6956 (2)	0.37501 (13)	0.56237 (12)	0.0456 (6)	
C30	0.6744 (3)	0.40074 (17)	0.63294 (14)	0.0672 (8)	
H30A	0.6129	0.4362	0.6283	0.101*	
H30B	0.7412	0.4247	0.6563	0.101*	
H30C	0.6558	0.3577	0.6590	0.101*	
C31	0.78362 (19)	0.32568 (12)	0.55509 (11)	0.0400 (5)	
H31	0.8299	0.3097	0.5945	0.048*	
C32	0.80654 (18)	0.29870 (11)	0.49148 (11)	0.0328 (5)	
C33	0.73323 (18)	0.31936 (11)	0.42974 (11)	0.0324 (5)	
C34	0.91051 (17)	0.25733 (11)	0.49200 (10)	0.0317 (5)	
H34	0.9559	0.2516	0.5342	0.038*	
C35	1.06421 (17)	0.19974 (11)	0.44743 (10)	0.0292 (4)	
C36	1.09688 (17)	0.15357 (11)	0.51390 (10)	0.0308 (4)	
C37	1.1988 (2)	0.16159 (13)	0.55531 (12)	0.0423 (5)	
H37	1.2496	0.1989	0.5462	0.051*	
C38	1.2248 (2)	0.11334 (14)	0.61061 (13)	0.0509 (6)	
H38	1.2935	0.1179	0.6392	0.061*	
C39	1.1493 (2)	0.05917 (13)	0.62305 (13)	0.0485 (6)	
H39	1.1648	0.0263	0.6602	0.058*	
C40	1.0494 (2)	0.05458 (13)	0.57885 (12)	0.0485 (6)	
H40	0.9979	0.0172	0.5869	0.058*	
C41	1.13974 (18)	0.27056 (11)	0.44456 (11)	0.0347 (5)	
C42	1.2027 (2)	0.28439 (15)	0.39317 (13)	0.0556 (7)	
H42	1.2023	0.2502	0.3571	0.067*	
C43	1.2670 (3)	0.34972 (18)	0.39518 (15)	0.0684 (8)	
H43	1.3112	0.3595	0.3609	0.082*	
C44	1.2651 (2)	0.39930 (15)	0.44755 (15)	0.0622 (8)	
H44	1.3085	0.4433	0.4504	0.075*	
C45	1.1978 (2)	0.38298 (13)	0.49609 (15)	0.0566 (7)	
H45	1.1949	0.4177	0.5314	0.068*	
C46	1.07506 (18)	0.14364 (11)	0.38840 (10)	0.0319 (5)	
C47	1.1755 (2)	0.10534 (13)	0.38571 (12)	0.0456 (6)	
H47	1.2375	0.1138	0.4191	0.055*	
C48	1.1829 (2)	0.05449 (14)	0.33312 (13)	0.0524 (7)	
H48	1.2502	0.0291	0.3301	0.063*	
C49	1.0890 (2)	0.04208 (12)	0.28530 (12)	0.0455 (6)	
H49	1.0912	0.0076	0.2497	0.055*	
C50	0.9919 (2)	0.08158 (11)	0.29106 (11)	0.0379 (5)	
H50	0.9285	0.0729	0.2587	0.045*	

### Atomic displacement parameters (Å^2^)

**Table:**

	*U*^11^	*U*^22^	*U*^33^	*U*^12^	*U*^13^	*U*^23^
Ni1	0.02992 (15)	0.02702 (13)	0.02723 (14)	−0.00083 (11)	0.00523 (11)	−0.00027 (10)
N1	0.0293 (9)	0.0294 (8)	0.0318 (9)	−0.0025 (7)	0.0051 (8)	0.0010 (7)
N2	0.0370 (10)	0.0295 (8)	0.0331 (9)	−0.0010 (8)	0.0082 (8)	0.0003 (7)
N3	0.0271 (9)	0.0276 (8)	0.0298 (9)	0.0016 (7)	0.0039 (7)	−0.0004 (7)
N4	0.0369 (10)	0.0272 (8)	0.0290 (9)	0.0003 (7)	0.0073 (8)	0.0014 (7)
N5	0.0427 (13)	0.0609 (13)	0.0803 (16)	0.0051 (11)	0.0246 (12)	0.0028 (12)
N6	0.0482 (12)	0.0378 (10)	0.0534 (12)	0.0048 (9)	0.0034 (10)	−0.0051 (9)
N7	0.0505 (13)	0.0360 (10)	0.0544 (12)	−0.0035 (9)	0.0088 (10)	−0.0049 (9)
N8	0.0421 (11)	0.0403 (10)	0.0407 (11)	−0.0063 (9)	0.0006 (9)	0.0052 (8)
O1	0.0417 (9)	0.0339 (7)	0.0361 (8)	−0.0063 (7)	0.0099 (7)	0.0008 (6)
O2	0.0379 (9)	0.0335 (7)	0.0332 (8)	0.0065 (6)	0.0057 (7)	0.0001 (6)
O3	0.0640 (13)	0.0591 (11)	0.0865 (14)	−0.0264 (10)	0.0211 (11)	0.0078 (10)
O4	0.0741 (15)	0.0912 (16)	0.0831 (15)	0.0470 (13)	0.0100 (12)	0.0144 (12)
C1	0.0406 (14)	0.0413 (13)	0.0606 (16)	−0.0032 (11)	0.0139 (12)	0.0087 (11)
C2	0.0312 (12)	0.0379 (12)	0.0451 (13)	0.0029 (9)	0.0081 (10)	0.0082 (10)
C3	0.0356 (13)	0.0496 (14)	0.0504 (14)	0.0025 (11)	0.0138 (11)	0.0159 (11)
C4	0.0397 (14)	0.0563 (14)	0.0388 (13)	0.0061 (11)	0.0138 (11)	0.0096 (11)
C5	0.0645 (18)	0.0784 (19)	0.0451 (15)	0.0028 (15)	0.0225 (14)	0.0079 (13)
C6	0.0400 (13)	0.0480 (13)	0.0359 (12)	0.0049 (10)	0.0077 (10)	0.0020 (10)
C7	0.0336 (12)	0.0367 (11)	0.0335 (11)	0.0006 (9)	0.0057 (10)	0.0037 (9)
C8	0.0304 (11)	0.0326 (10)	0.0355 (11)	0.0045 (9)	0.0069 (9)	0.0080 (9)
C9	0.0353 (12)	0.0356 (11)	0.0327 (11)	−0.0023 (9)	0.0038 (10)	−0.0010 (9)
C10	0.0303 (11)	0.0309 (10)	0.0347 (11)	−0.0048 (9)	0.0047 (9)	−0.0023 (8)
C11	0.0327 (12)	0.0337 (11)	0.0358 (12)	−0.0055 (9)	0.0062 (10)	−0.0022 (9)
C12	0.0487 (15)	0.0461 (13)	0.0507 (15)	0.0044 (11)	−0.0036 (13)	−0.0072 (11)
C13	0.0613 (18)	0.0700 (18)	0.0501 (16)	−0.0103 (15)	−0.0030 (14)	−0.0194 (14)
C14	0.0682 (19)	0.0455 (15)	0.0621 (18)	−0.0089 (14)	0.0153 (15)	−0.0226 (13)
C15	0.0656 (18)	0.0377 (13)	0.0667 (17)	0.0044 (12)	0.0166 (15)	−0.0106 (12)
C16	0.0360 (12)	0.0374 (11)	0.0339 (11)	−0.0006 (9)	0.0039 (10)	−0.0059 (9)
C17	0.0390 (14)	0.0523 (15)	0.0699 (18)	0.0051 (12)	0.0080 (13)	0.0138 (13)
C18	0.064 (2)	0.0564 (16)	0.074 (2)	0.0173 (15)	0.0054 (16)	0.0168 (14)
C19	0.0514 (18)	0.076 (2)	0.0645 (18)	0.0269 (15)	0.0055 (15)	−0.0054 (15)
C20	0.0457 (17)	0.084 (2)	0.089 (2)	0.0133 (15)	0.0261 (16)	−0.0004 (18)
C21	0.0371 (13)	0.0302 (10)	0.0365 (12)	−0.0026 (9)	0.0082 (10)	−0.0026 (9)
C22	0.0445 (15)	0.0488 (13)	0.0480 (14)	−0.0151 (11)	0.0082 (12)	−0.0017 (11)
C23	0.0535 (16)	0.0509 (14)	0.0569 (16)	−0.0174 (12)	0.0181 (14)	0.0068 (12)
C24	0.0589 (17)	0.0487 (14)	0.0445 (14)	−0.0056 (12)	0.0164 (13)	0.0119 (11)
C25	0.0454 (14)	0.0388 (12)	0.0380 (12)	−0.0018 (10)	0.0075 (11)	0.0040 (10)
C26	0.0485 (16)	0.0623 (16)	0.0556 (16)	0.0215 (13)	0.0066 (13)	0.0036 (13)
C27	0.0378 (13)	0.0356 (11)	0.0469 (13)	0.0063 (10)	0.0101 (11)	0.0004 (10)
C28	0.0428 (14)	0.0439 (13)	0.0569 (16)	0.0121 (11)	0.0129 (12)	−0.0059 (11)
C29	0.0493 (15)	0.0471 (13)	0.0427 (13)	0.0078 (11)	0.0149 (12)	−0.0090 (11)
C30	0.077 (2)	0.0749 (19)	0.0530 (16)	0.0255 (16)	0.0197 (15)	−0.0155 (14)
C31	0.0406 (13)	0.0445 (12)	0.0356 (12)	0.0033 (10)	0.0074 (10)	−0.0047 (10)
C32	0.0322 (12)	0.0323 (10)	0.0347 (11)	0.0015 (9)	0.0078 (9)	−0.0020 (9)
C33	0.0316 (12)	0.0285 (10)	0.0383 (12)	−0.0012 (9)	0.0090 (10)	−0.0006 (9)
C34	0.0326 (12)	0.0330 (10)	0.0291 (11)	0.0012 (9)	0.0032 (9)	−0.0012 (8)
C35	0.0273 (11)	0.0321 (10)	0.0280 (10)	0.0028 (8)	0.0037 (9)	−0.0021 (8)
C36	0.0321 (12)	0.0283 (10)	0.0320 (11)	0.0033 (9)	0.0047 (9)	−0.0021 (8)
C37	0.0359 (13)	0.0397 (12)	0.0489 (14)	−0.0025 (10)	−0.0024 (11)	0.0058 (10)
C38	0.0475 (15)	0.0483 (14)	0.0518 (15)	0.0030 (12)	−0.0109 (12)	0.0037 (12)
C39	0.0596 (17)	0.0418 (13)	0.0420 (13)	0.0083 (12)	0.0001 (13)	0.0094 (11)
C40	0.0584 (17)	0.0410 (13)	0.0457 (14)	−0.0079 (12)	0.0057 (13)	0.0104 (11)
C41	0.0325 (12)	0.0326 (11)	0.0381 (12)	0.0012 (9)	0.0020 (10)	0.0035 (9)
C42	0.0593 (17)	0.0626 (16)	0.0478 (15)	−0.0202 (14)	0.0175 (13)	−0.0089 (12)
C43	0.066 (2)	0.081 (2)	0.0605 (18)	−0.0323 (16)	0.0190 (15)	0.0057 (16)
C44	0.0618 (18)	0.0476 (15)	0.074 (2)	−0.0203 (13)	−0.0023 (16)	0.0105 (14)
C45	0.0627 (18)	0.0355 (13)	0.0703 (18)	−0.0051 (12)	0.0046 (15)	−0.0073 (12)
C46	0.0354 (12)	0.0302 (10)	0.0320 (11)	0.0021 (9)	0.0110 (10)	0.0012 (8)
C47	0.0393 (14)	0.0524 (14)	0.0452 (14)	0.0117 (11)	0.0065 (11)	−0.0051 (11)
C48	0.0536 (17)	0.0533 (15)	0.0537 (16)	0.0179 (12)	0.0197 (14)	−0.0018 (12)
C49	0.0667 (18)	0.0359 (12)	0.0372 (13)	0.0077 (11)	0.0196 (13)	−0.0040 (10)
C50	0.0525 (15)	0.0306 (11)	0.0310 (11)	0.0013 (10)	0.0075 (11)	−0.0007 (9)

### Geometric parameters (Å, °)

**Table:**

Ni1—N3	2.0210 (17)	C18—C19	1.346 (4)
Ni1—O2	2.0283 (13)	C18—H18	0.9300
Ni1—N1	2.0318 (17)	C19—C20	1.366 (4)
Ni1—O1	2.0322 (13)	C19—H19	0.9300
Ni1—N2	2.1084 (16)	C20—H20	0.9300
Ni1—N4	2.1308 (16)	C21—C22	1.390 (3)
N1—C9	1.291 (2)	C22—C23	1.378 (3)
N1—C10	1.486 (2)	C22—H22	0.9300
N2—C21	1.335 (3)	C23—C24	1.375 (4)
N2—C25	1.353 (3)	C23—H23	0.9300
N3—C34	1.288 (2)	C24—C25	1.371 (3)
N3—C35	1.480 (2)	C24—H24	0.9300
N4—C46	1.336 (3)	C25—H25	0.9300
N4—C50	1.352 (3)	C26—C27	1.442 (3)
N5—C16	1.333 (3)	C26—H26	0.9300
N5—C20	1.335 (4)	C27—C28	1.392 (3)
N6—C11	1.337 (3)	C27—C33	1.437 (3)
N6—C15	1.347 (3)	C28—C29	1.373 (3)
N7—C41	1.335 (3)	C28—H28	0.9300
N7—C45	1.341 (3)	C29—C31	1.389 (3)
N8—C36	1.333 (3)	C29—C30	1.515 (3)
N8—C40	1.336 (3)	C30—H30A	0.9600
O1—C8	1.281 (2)	C30—H30B	0.9600
O2—C33	1.285 (2)	C30—H30C	0.9600
O3—C1	1.216 (3)	C31—C32	1.401 (3)
O4—C26	1.210 (3)	C31—H31	0.9300
C1—C2	1.450 (3)	C32—C33	1.439 (3)
C1—H1	0.9300	C32—C34	1.440 (3)
C2—C3	1.386 (3)	C34—H34	0.9300
C2—C8	1.439 (3)	C35—C36	1.542 (3)
C3—C4	1.382 (3)	C35—C46	1.545 (3)
C3—H3	0.9300	C35—C41	1.547 (3)
C4—C6	1.390 (3)	C36—C37	1.373 (3)
C4—C5	1.516 (3)	C37—C38	1.381 (3)
C5—H5A	0.9600	C37—H37	0.9300
C5—H5B	0.9600	C38—C39	1.360 (3)
C5—H5C	0.9600	C38—H38	0.9300
C6—C7	1.405 (3)	C39—C40	1.376 (3)
C6—H6	0.9300	C39—H39	0.9300
C7—C8	1.442 (3)	C40—H40	0.9300
C7—C9	1.443 (3)	C41—C42	1.366 (3)
C9—H9	0.9300	C42—C43	1.382 (4)
C10—C11	1.540 (3)	C42—H42	0.9300
C10—C21	1.540 (3)	C43—C44	1.353 (4)
C10—C16	1.543 (3)	C43—H43	0.9300
C11—C12	1.373 (3)	C44—C45	1.364 (4)
C12—C13	1.386 (3)	C44—H44	0.9300
C12—H12	0.9300	C45—H45	0.9300
C13—C14	1.358 (4)	C46—C47	1.386 (3)
C13—H13	0.9300	C47—C48	1.381 (3)
C14—C15	1.363 (4)	C47—H47	0.9300
C14—H14	0.9300	C48—C49	1.376 (4)
C15—H15	0.9300	C48—H48	0.9300
C16—C17	1.370 (3)	C49—C50	1.372 (3)
C17—C18	1.384 (4)	C49—H49	0.9300
C17—H17	0.9300	C50—H50	0.9300
			
N3—Ni1—O2	89.66 (6)	C19—C20—H20	117.9
N3—Ni1—N1	173.34 (6)	N2—C21—C22	121.9 (2)
O2—Ni1—N1	95.57 (6)	N2—C21—C10	116.98 (17)
N3—Ni1—O1	94.07 (6)	C22—C21—C10	121.1 (2)
O2—Ni1—O1	90.84 (6)	C23—C22—C21	118.9 (2)
N1—Ni1—O1	89.98 (6)	C23—C22—H22	120.6
N3—Ni1—N2	97.48 (7)	C21—C22—H22	120.6
O2—Ni1—N2	89.40 (6)	C24—C23—C22	119.4 (2)
N1—Ni1—N2	78.51 (6)	C24—C23—H23	120.3
O1—Ni1—N2	168.45 (6)	C22—C23—H23	120.3
N3—Ni1—N4	78.83 (6)	C25—C24—C23	118.9 (2)
O2—Ni1—N4	168.03 (6)	C25—C24—H24	120.6
N1—Ni1—N4	96.14 (7)	C23—C24—H24	120.6
O1—Ni1—N4	86.73 (6)	N2—C25—C24	122.5 (2)
N2—Ni1—N4	95.32 (6)	N2—C25—H25	118.7
C9—N1—C10	119.83 (18)	C24—C25—H25	118.7
C9—N1—Ni1	123.35 (15)	O4—C26—C27	126.1 (3)
C10—N1—Ni1	116.58 (12)	O4—C26—H26	117.0
C21—N2—C25	118.38 (18)	C27—C26—H26	117.0
C21—N2—Ni1	116.07 (13)	C28—C27—C33	120.7 (2)
C25—N2—Ni1	125.48 (15)	C28—C27—C26	120.3 (2)
C34—N3—C35	117.97 (17)	C33—C27—C26	119.0 (2)
C34—N3—Ni1	124.40 (14)	C29—C28—C27	123.3 (2)
C35—N3—Ni1	117.51 (12)	C29—C28—H28	118.3
C46—N4—C50	117.99 (18)	C27—C28—H28	118.3
C46—N4—Ni1	114.47 (12)	C28—C29—C31	116.7 (2)
C50—N4—Ni1	126.93 (15)	C28—C29—C30	122.7 (2)
C16—N5—C20	117.8 (2)	C31—C29—C30	120.7 (2)
C11—N6—C15	117.2 (2)	C29—C30—H30A	109.5
C41—N7—C45	117.6 (2)	C29—C30—H30B	109.5
C36—N8—C40	117.5 (2)	H30A—C30—H30B	109.5
C8—O1—Ni1	125.87 (13)	C29—C30—H30C	109.5
C33—O2—Ni1	126.68 (13)	H30A—C30—H30C	109.5
O3—C1—C2	125.0 (2)	H30B—C30—H30C	109.5
O3—C1—H1	117.5	C29—C31—C32	123.5 (2)
C2—C1—H1	117.5	C29—C31—H31	118.2
C3—C2—C8	121.3 (2)	C32—C31—H31	118.2
C3—C2—C1	119.4 (2)	C31—C32—C33	119.59 (19)
C8—C2—C1	119.3 (2)	C31—C32—C34	116.4 (2)
C4—C3—C2	123.2 (2)	C33—C32—C34	123.74 (18)
C4—C3—H3	118.4	O2—C33—C27	120.2 (2)
C2—C3—H3	118.4	O2—C33—C32	123.87 (18)
C3—C4—C6	116.5 (2)	C27—C33—C32	115.92 (18)
C3—C4—C5	121.9 (2)	N3—C34—C32	125.9 (2)
C6—C4—C5	121.5 (2)	N3—C34—H34	117.1
C4—C5—H5A	109.5	C32—C34—H34	117.1
C4—C5—H5B	109.5	N3—C35—C36	112.89 (15)
H5A—C5—H5B	109.5	N3—C35—C46	108.19 (16)
C4—C5—H5C	109.5	C36—C35—C46	105.11 (15)
H5A—C5—H5C	109.5	N3—C35—C41	106.29 (15)
H5B—C5—H5C	109.5	C36—C35—C41	112.23 (17)
C4—C6—C7	123.7 (2)	C46—C35—C41	112.17 (16)
C4—C6—H6	118.2	N8—C36—C37	122.31 (19)
C7—C6—H6	118.2	N8—C36—C35	114.50 (18)
C6—C7—C8	119.54 (19)	C37—C36—C35	122.98 (18)
C6—C7—C9	116.1 (2)	C36—C37—C38	119.0 (2)
C8—C7—C9	123.95 (18)	C36—C37—H37	120.5
O1—C8—C2	120.34 (19)	C38—C37—H37	120.5
O1—C8—C7	123.94 (18)	C39—C38—C37	119.6 (2)
C2—C8—C7	115.71 (18)	C39—C38—H38	120.2
N1—C9—C7	126.0 (2)	C37—C38—H38	120.2
N1—C9—H9	117.0	C38—C39—C40	117.8 (2)
C7—C9—H9	117.0	C38—C39—H39	121.1
N1—C10—C11	112.20 (16)	C40—C39—H39	121.1
N1—C10—C21	107.70 (16)	N8—C40—C39	123.8 (2)
C11—C10—C21	108.25 (16)	N8—C40—H40	118.1
N1—C10—C16	108.91 (16)	C39—C40—H40	118.1
C11—C10—C16	110.35 (17)	N7—C41—C42	121.8 (2)
C21—C10—C16	109.36 (16)	N7—C41—C35	114.29 (18)
N6—C11—C12	122.2 (2)	C42—C41—C35	123.9 (2)
N6—C11—C10	114.37 (19)	C41—C42—C43	119.4 (2)
C12—C11—C10	123.5 (2)	C41—C42—H42	120.3
C11—C12—C13	119.1 (2)	C43—C42—H42	120.3
C11—C12—H12	120.4	C44—C43—C42	119.4 (3)
C13—C12—H12	120.4	C44—C43—H43	120.3
C14—C13—C12	119.2 (3)	C42—C43—H43	120.3
C14—C13—H13	120.4	C43—C44—C45	118.2 (2)
C12—C13—H13	120.4	C43—C44—H44	120.9
C13—C14—C15	118.5 (2)	C45—C44—H44	120.9
C13—C14—H14	120.7	N7—C45—C44	123.7 (2)
C15—C14—H14	120.7	N7—C45—H45	118.2
N6—C15—C14	123.7 (2)	C44—C45—H45	118.2
N6—C15—H15	118.1	N4—C46—C47	121.82 (19)
C14—C15—H15	118.1	N4—C46—C35	117.70 (17)
N5—C16—C17	121.5 (2)	C47—C46—C35	120.5 (2)
N5—C16—C10	114.86 (19)	C48—C47—C46	119.6 (2)
C17—C16—C10	123.5 (2)	C48—C47—H47	120.2
C16—C17—C18	118.9 (2)	C46—C47—H47	120.2
C16—C17—H17	120.5	C49—C48—C47	118.8 (2)
C18—C17—H17	120.5	C49—C48—H48	120.6
C19—C18—C17	120.3 (3)	C47—C48—H48	120.6
C19—C18—H18	119.8	C50—C49—C48	118.7 (2)
C17—C18—H18	119.8	C50—C49—H49	120.6
C18—C19—C20	117.3 (3)	C48—C49—H49	120.6
C18—C19—H19	121.3	N4—C50—C49	123.1 (2)
C20—C19—H19	121.3	N4—C50—H50	118.5
N5—C20—C19	124.2 (3)	C49—C50—H50	118.5
N5—C20—H20	117.9		
			
O2—Ni1—N1—C9	116.19 (16)	N5—C16—C17—C18	−0.6 (4)
O1—Ni1—N1—C9	25.34 (17)	C10—C16—C17—C18	175.0 (2)
N2—Ni1—N1—C9	−155.57 (17)	C16—C17—C18—C19	0.6 (4)
N4—Ni1—N1—C9	−61.37 (17)	C17—C18—C19—C20	−0.8 (5)
O2—Ni1—N1—C10	−69.48 (13)	C16—N5—C20—C19	−0.8 (5)
O1—Ni1—N1—C10	−160.32 (13)	C18—C19—C20—N5	0.9 (5)
N2—Ni1—N1—C10	18.77 (13)	C25—N2—C21—C22	−0.4 (3)
N4—Ni1—N1—C10	112.97 (13)	Ni1—N2—C21—C22	−177.35 (16)
N3—Ni1—N2—C21	173.56 (14)	C25—N2—C21—C10	179.95 (18)
O2—Ni1—N2—C21	83.98 (14)	Ni1—N2—C21—C10	3.0 (2)
N1—Ni1—N2—C21	−11.83 (14)	N1—C10—C21—N2	11.5 (2)
O1—Ni1—N2—C21	−7.3 (4)	C11—C10—C21—N2	133.05 (19)
N4—Ni1—N2—C21	−107.04 (14)	C16—C10—C21—N2	−106.7 (2)
N3—Ni1—N2—C25	−3.14 (17)	N1—C10—C21—C22	−168.14 (19)
O2—Ni1—N2—C25	−92.72 (17)	C11—C10—C21—C22	−46.6 (3)
N1—Ni1—N2—C25	171.47 (18)	C16—C10—C21—C22	73.7 (2)
O1—Ni1—N2—C25	176.0 (3)	N2—C21—C22—C23	1.2 (3)
N4—Ni1—N2—C25	76.27 (17)	C10—C21—C22—C23	−179.1 (2)
O2—Ni1—N3—C34	23.99 (16)	C21—C22—C23—C24	−0.9 (4)
O1—Ni1—N3—C34	114.82 (16)	C22—C23—C24—C25	−0.1 (4)
N2—Ni1—N3—C34	−65.35 (16)	C21—N2—C25—C24	−0.7 (3)
N4—Ni1—N3—C34	−159.33 (17)	Ni1—N2—C25—C24	175.90 (17)
O2—Ni1—N3—C35	−160.07 (13)	C23—C24—C25—N2	1.0 (4)
O1—Ni1—N3—C35	−69.24 (13)	O4—C26—C27—C28	1.5 (4)
N2—Ni1—N3—C35	110.59 (13)	O4—C26—C27—C33	−178.3 (3)
N4—Ni1—N3—C35	16.61 (12)	C33—C27—C28—C29	−0.1 (4)
N3—Ni1—N4—C46	−14.76 (13)	C26—C27—C28—C29	−179.9 (2)
O2—Ni1—N4—C46	1.5 (4)	C27—C28—C29—C31	3.4 (4)
N1—Ni1—N4—C46	169.66 (13)	C27—C28—C29—C30	−177.3 (2)
O1—Ni1—N4—C46	80.04 (13)	C28—C29—C31—C32	−1.8 (4)
N2—Ni1—N4—C46	−111.36 (14)	C30—C29—C31—C32	178.9 (2)
N3—Ni1—N4—C50	174.48 (17)	C29—C31—C32—C33	−3.1 (3)
O2—Ni1—N4—C50	−169.3 (2)	C29—C31—C32—C34	170.9 (2)
N1—Ni1—N4—C50	−1.09 (17)	Ni1—O2—C33—C27	−173.70 (14)
O1—Ni1—N4—C50	−90.72 (16)	Ni1—O2—C33—C32	8.2 (3)
N2—Ni1—N4—C50	77.88 (17)	C28—C27—C33—O2	177.1 (2)
N3—Ni1—O1—C8	150.46 (16)	C26—C27—C33—O2	−3.1 (3)
O2—Ni1—O1—C8	−119.82 (16)	C28—C27—C33—C32	−4.7 (3)
N1—Ni1—O1—C8	−24.24 (16)	C26—C27—C33—C32	175.1 (2)
N2—Ni1—O1—C8	−28.7 (4)	C31—C32—C33—O2	−175.76 (19)
N4—Ni1—O1—C8	71.91 (16)	C34—C32—C33—O2	10.7 (3)
N3—Ni1—O2—C33	−21.03 (16)	C31—C32—C33—C27	6.1 (3)
N1—Ni1—O2—C33	154.85 (16)	C34—C32—C33—C27	−167.43 (19)
O1—Ni1—O2—C33	−115.09 (16)	C35—N3—C34—C32	168.85 (18)
N2—Ni1—O2—C33	76.46 (16)	Ni1—N3—C34—C32	−15.2 (3)
N4—Ni1—O2—C33	−37.0 (4)	C31—C32—C34—N3	179.5 (2)
O3—C1—C2—C3	−3.9 (4)	C33—C32—C34—N3	−6.8 (3)
O3—C1—C2—C8	179.7 (2)	C34—N3—C35—C36	44.9 (2)
C8—C2—C3—C4	0.2 (3)	Ni1—N3—C35—C36	−131.33 (14)
C1—C2—C3—C4	−176.1 (2)	C34—N3—C35—C46	160.76 (17)
C2—C3—C4—C6	0.2 (3)	Ni1—N3—C35—C46	−15.45 (19)
C2—C3—C4—C5	175.7 (2)	C34—N3—C35—C41	−78.6 (2)
C3—C4—C6—C7	1.6 (3)	Ni1—N3—C35—C41	105.21 (15)
C5—C4—C6—C7	−173.8 (2)	C40—N8—C36—C37	0.0 (3)
C4—C6—C7—C8	−3.8 (3)	C40—N8—C36—C35	174.74 (19)
C4—C6—C7—C9	168.7 (2)	N3—C35—C36—N8	47.8 (2)
Ni1—O1—C8—C2	−170.29 (14)	C46—C35—C36—N8	−69.9 (2)
Ni1—O1—C8—C7	11.2 (3)	C41—C35—C36—N8	167.90 (17)
C3—C2—C8—O1	179.1 (2)	N3—C35—C36—C37	−137.5 (2)
C1—C2—C8—O1	−4.6 (3)	C46—C35—C36—C37	104.8 (2)
C3—C2—C8—C7	−2.3 (3)	C41—C35—C36—C37	−17.4 (3)
C1—C2—C8—C7	174.02 (19)	N8—C36—C37—C38	−0.1 (3)
C6—C7—C8—O1	−177.48 (19)	C35—C36—C37—C38	−174.5 (2)
C9—C7—C8—O1	10.6 (3)	C36—C37—C38—C39	−0.1 (4)
C6—C7—C8—C2	3.9 (3)	C37—C38—C39—C40	0.5 (4)
C9—C7—C8—C2	−168.02 (19)	C36—N8—C40—C39	0.5 (4)
C10—N1—C9—C7	171.24 (19)	C38—C39—C40—N8	−0.7 (4)
Ni1—N1—C9—C7	−14.6 (3)	C45—N7—C41—C42	−1.3 (4)
C6—C7—C9—N1	179.3 (2)	C45—N7—C41—C35	−179.0 (2)
C8—C7—C9—N1	−8.5 (3)	N3—C35—C41—N7	63.8 (2)
C9—N1—C10—C11	33.8 (3)	C36—C35—C41—N7	−60.0 (2)
Ni1—N1—C10—C11	−140.70 (14)	C46—C35—C41—N7	−178.10 (19)
C9—N1—C10—C21	152.88 (18)	N3—C35—C41—C42	−113.8 (2)
Ni1—N1—C10—C21	−21.66 (19)	C36—C35—C41—C42	122.3 (2)
C9—N1—C10—C16	−88.6 (2)	C46—C35—C41—C42	4.2 (3)
Ni1—N1—C10—C16	96.83 (16)	N7—C41—C42—C43	2.1 (4)
C15—N6—C11—C12	−2.7 (3)	C35—C41—C42—C43	179.6 (2)
C15—N6—C11—C10	177.8 (2)	C41—C42—C43—C44	−0.9 (5)
N1—C10—C11—N6	70.4 (2)	C42—C43—C44—C45	−0.9 (5)
C21—C10—C11—N6	−48.3 (2)	C41—N7—C45—C44	−0.7 (4)
C16—C10—C11—N6	−167.90 (18)	C43—C44—C45—N7	1.8 (4)
N1—C10—C11—C12	−109.1 (2)	C50—N4—C46—C47	0.8 (3)
C21—C10—C11—C12	132.2 (2)	Ni1—N4—C46—C47	−170.80 (16)
C16—C10—C11—C12	12.6 (3)	C50—N4—C46—C35	−177.78 (17)
N6—C11—C12—C13	2.0 (4)	Ni1—N4—C46—C35	10.6 (2)
C10—C11—C12—C13	−178.6 (2)	N3—C35—C46—N4	2.4 (2)
C11—C12—C13—C14	0.0 (4)	C36—C35—C46—N4	123.30 (18)
C12—C13—C14—C15	−1.1 (4)	C41—C35—C46—N4	−114.48 (19)
C11—N6—C15—C14	1.5 (4)	N3—C35—C46—C47	−176.19 (18)
C13—C14—C15—N6	0.3 (4)	C36—C35—C46—C47	−55.3 (2)
C20—N5—C16—C17	0.7 (4)	C41—C35—C46—C47	66.9 (2)
C20—N5—C16—C10	−175.3 (2)	N4—C46—C47—C48	0.4 (3)
N1—C10—C16—N5	−167.85 (19)	C35—C46—C47—C48	179.0 (2)
C11—C10—C16—N5	68.6 (2)	C46—C47—C48—C49	−1.3 (4)
C21—C10—C16—N5	−50.4 (2)	C47—C48—C49—C50	1.0 (4)
N1—C10—C16—C17	16.3 (3)	C46—N4—C50—C49	−1.2 (3)
C11—C10—C16—C17	−107.3 (2)	Ni1—N4—C50—C49	169.31 (16)
C21—C10—C16—C17	133.7 (2)	C48—C49—C50—N4	0.2 (3)
